# Horse Doctors

**Published:** 1873-05

**Authors:** 


### HORSE DOCTORS.

In the progress of men and things, the medical treatment of the diseases of domestic animals is becoming more and more of a science. The brute creation is liable to many of the diseases which affect man, and these can be successfully treated on the same general principles, and in many cases, with the same or similar remedies. Every thrifty and intelligent farmer would do well to obtain a five dollar book, of over seven hundred pages, published by Boericke and Tafel, of New York and Philadelphia, entitled "A Manual of Homeopathic Veterinary Practice, designed for horses and all kinds of domestic animals and fowls, prescribing their proper treatment when injured or diseased, and their particular care and general management and health." It is not intended or expected that the horses, cows and roosters will read the book, but that the owners shall study it and thereby save thousands of dollars a year.

Cornelius Vanderbilt, quite unwittingly, the other day, carried away the heart of a New York lady, thus: The weather was piercingly cold; he drove up to his Grand Union depot, in Forty-second Street, secured the horse, and then carefully spread over him a warm blanket. Such thoughtfulness is worthy of recommendation.

The preservation of the health of domestic animals can be studied to great advantage, and the manual referred to is full of suggestions in reference to the early symptoms of animal diseases, the remedies used and the modes of cure. There are not a few who take more pains to preserve the health of a favorite horse, a poodle dog or Maltese kitten, than their own, or that of their wives and children.

